# Physical activity and sperm quality: influence in sperm donors

**DOI:** 10.1186/s12958-022-00946-x

**Published:** 2022-05-24

**Authors:** Roberto Matorras, Alfredo Navarro, Dagoberto Ramos, Iker Malaina, Jon Irazusta, Alberto Vendrell, Amaia Fernandez, Marcos Ferrando, Fernando Quintana

**Affiliations:** 1 IVIRMA BILBAO, Lejona, Spain; 2 Obstetrics and Gynecology Department, Department of Medical-Surgical Specialties, Cruces University Hospital, Basque Country University, Baracaldo, Spain; 3 Biocruces Health Research Institute, Baracaldo, Spain; 4 IVI Foundation, Valencia, Spain; 5 Department of Mathematics, Basque Country University, Lejona, Spain; 6 Department of Physiology, Basque Country University, Lejona, Spain

**Keywords:** Physical activity, IPAQ, Spermiogram, IVF, Artificial insemination donor, Sperm quality, Sport

## Abstract

**Purpose:**

To ascertain whether physical activity (PA) is associated with better sperm quality in sperm donors.

**Methods:**

A prospective case–control study was designed in an IVF center setting. A total of 207 sperm donation candidates from a relatively small geographical area were included in the study with no intervention.

Donor candidates were subjected to conventional sperm analysis according to WHO criteria. Moreover, they answered a standardized questionnaire regarding their last week PA (IPAQ), with PA expressed in metabolic equivalents (METs)-min/week. Donors were classified into 4 groups: low, moderate, high and very high PA. Specific sports were included in the questionnaire. Semen samples from 43 accepted donors were used in artificial insemination by donor (AID) and IVF. The fertilization rates (FR) and pregnancy rates (PR) were studied.

**Results:**

Semen volume, sperm concentration, progressive spermatozoa, non-progressive spermatozoa, total motile progressive spermatozoa and sperm morphology were similar in the four PA groups. No correlation between various semen parameters studied and METs was found. Running or cycling > 1 h/week did not influence sperm parameters. The AID PR was similar in the different PA groups. However, in IVF the mean donor FR was significantly higher in the high PA group and in the very high PA group.

**Conclusions:**

No detrimental effect was associated with PA, or even very high PA, regarding conventional sperm parameters. Moreover, a better FR was associated with high and very high PA in IVF cycles, which merits more studies.

**Supplementary Information:**

The online version contains supplementary material available at 10.1186/s12958-022-00946-x.

## Introduction

Physical activity (PA) has a number of beneficial effects on health. Its preventive impact on hypertension, cardiovascular disorders, diabetes, obesity and depression, among other conditions, is well known [9, 19, 27, 52]. It has been reported that regular PA extends the average lifespan by 6–7 years [9, 19, 27, 52].

When it comes to infertility, increasing attention is being paid to promoting healthy habits in both men and women, as it is well known that weight problems, smoking and alcohol, among other factors, are detrimental to fertility [3, 11, 53]. However, Regarding PA, it is well known that excessive PA is associated with amenorrhea [18] and there are some recommendations concerning the PA to be taken up during pregnancy [4]. However there are no guidelines regarding the PA for men in infertile couples, and the ideal level of PA in male partners of infertile couples, if any, is unknown.

Some authors have reported no effect of PA [41] whereas others have reported impaired sperm quality in men with lower PA [58] whereas impaired sperm quality has been reported among athletes with very high PA [57, 60].

Several mechanisms have been proposed by which PA may influence seminal quality. Regular exercise could decrease oxidative stress by promoting antioxidant enzyme activity and upregulating endogenous antioxidant defense [20, 48, 49, 56]. On the other hand, high-intensity PA could alter the oxidant/antioxidant balance [16]. In addition, there are controversial reports on hormonal changes at the hypothalamic, testicular and adrenal levels as a function of PA intensity [39].

However, there are many clinical and demographic parameters that can be linked both with PA and sperm quality such as youth, socioeconomic status, healthy diet and health status. On the other hand, obesity, smoking, drug consumption, alcohol and a number of health conditions, all of them associated with impaired sperm quality [24, 25, 54, 61] are less frequent in physically active men. Another constraint of previous reports is population recruitment. Since sperm analysis is seldom performed in unselected populations, most studies center on infertile patients or on men practicing a sport, or even elite athletes [14, 32, 33, 59, 62]. Therefore, we have focused our study on a population which shares a number of characteristics with the unselected demographic: sperm donors.

Moreover, most previous studies have considered only the sportive PA, ignoring non-recreational activities such as walking, which can represent a considerable proportion of daily PA. Non-recreational activity is covered in the IPAQ [23] which is the method we used.

Finally, there is general agreement that the WHO classical sperm analysis has its limitations as a predictor of fertility status [36, 42]. A number of authors have highlighted oxidative stress as a major cause of male infertility but there is no agreement concerning the best method to assess it or the cutoff points of normality [15, 36]. Moreover, while the best marker of good sperm quality is a natural pregnancy, the absence of pregnancy does not imply that sperm quality is poor, since it could be due to concomitant female factors.

Since PA remains relatively stable over time [46], the study of PA during a randomly selected week could give good information on PA during the last months, where the most important steps of spermatogenesis take place. Therefore, the aim of our study was to evaluate whether PA performed 1 week before sperm analysis could be an indicator of sperm quality.

In our study, we have included not only classical sperm parameters, but also PR rates in AID cycles and fertilization rates in IVF cycles, although it is controversial whether high quality sperm samples in ART produce higher PR than normal-quality sperm samples.

## Material and Methods

The population under study consisted of 207 potential donor candidates at the IVI Bilbao assisted reproduction clinic (Basque Country, Spain) from September 2015 to June 2018. During that period, all the candidates applying to be a sperm donor at our clinic were invited to participate in the study. All of them came from our geographical area, which has about 1,110,000 inhabitants and covers 2200 km^2^. In our country, sperm donation is anonymous, and although payment is not permitted, the law allows a compensatory reimbursement.

The study was approved by our Institutional Review Board (CEIC Euskadi PI2015033) and it was registered on ClinicalTrials.gov (Identifier: NCT02683057). All the participants signed a written consent to participate in the study.

During the first visit, all of the potential donors underwent a psychophysical examination and also completed a questionnaire concerning demographics, medical and reproductive history, medication (antibiotics, antidepressants, hormones and illicit drug consumption) and smoking habits. The examinations performed were a physical examination, biometric measurement, glucose analysis, hematocrit and hemoglobin measurement, and sperm analysis. The main characteristics of sperm donation candidates are given in Table 1. They were specifically asked about their PA. No comments were made to the potential donors concerning the hypothetical effects of PA on sperm quality. Their PA was researched by means of the IPAQ test [23].

**Table 1:** Demographic characteristics of sperm donation candidates

	**Mean **± **SD**	**Median (IC95_inf - IC95_sup)**
**Age (years)**	23.23 ± 4.61	22.0 (22.6 - 23.87)
**Weight (kg)**	72.71 ± 8.84	71.0 (74.47 - 73.96)
**Height (m)**	1.77 ±0.06	1.77 (1.76 - 1.78)
**BMI (kg/m2)**	23.24 ± 2.35	23.3 (22.91 - 23.57)
**Glucose (mg/dL)**	93.60 ± 13.09	91.0 (91.23 - 95.97)
**Hematocrit (%)**	44.98 ± 4.21	45.1 (44.22 - 45.75)
**Hemoglobin (g/dL)**	15.25 ± 2.80	15.0 (14.74 - 15.75)
	**%**	
**University students**	43 (89/207)	
**Smoking**	22.7 (47/207)	
**occasional regular vs Occasional-regular alcohol consumption**	81.8 (169/207)	
**alcohol consumption vs Occasional illicit drug use**	6.8 (14/207)	

The IPAQ is a standardized and internationally validated method that evaluates the physical activity performed in the last week by means of a questionnaire. It has been shown to correlate well with physical activity performed in the preceding weeks and with that performed in a typical week [12, 56], as well as with that measured by accelerometers [45].

The methodology applied has been described previously [22]. On the day of the sperm sample collection, donors were requested to fill out the short version of the IPAQ. The purpose of the questionnaire was to gather information on the participants’ PA during the previous week in terms of frequency (days per week) and duration (minutes per week), and this was classified into three categories according to intensity: vigorous PA, moderate PA, or walking activities [23]. Vigorous PA corresponds to activities that take hard physical effort and make breathe much harder than normal like heavy aerobics, lifting, digging, or fast bicycling. Moderate PA refer to activities that take moderate physical effort and make breathe somewhat harder than normal, like bicycling at a regular pace, carrying light loads, or doubles tennis. In vigorous and moderate PA only those PA done for at least 10 min at a time are taken into account. Light activity corresponds to walking (at work, at home, walking to travel from place to place, and any other walking done solely for recreation or sport) [23]. The intensity of the PA was estimated using metabolic equivalents (METs). For each level of physical activity, the average METs were calculated using the compendium by Ainsworth [2] attributing 8 METs for Vigorous PA, 4 METs for Moderate PA and 3.3 METs for Walking. Using these measurements, the results of the short version of the IPAQ were calculated, obtaining a total PA score in METs-min/week. Following the IPAQ indications, individuals were classified according to their total PA scores into three groups from lowest to highest PA intensity. In addition, the group with the highest intensity was divided into two subgroups, obtaining a classification of four intensities according to its result in MET-min/week: low (≤ 599), moderate (600–2999), high (3000–5999) and very high (≥ 6000). Participants reported which sports they practiced and how many hours they dedicated to each of them during the week before answering the questionnaire.

Sperm analysis was carried out blind, without knowing the IPAQ results, by the same investigator (FQ). All samples were produced after 3–5 days of sexual abstinence.

Sperm analysis was performed as follows. Ejaculates were analyzed according to the World Health Organization (WHO) manual for examining and processing human semen [63]. Concentrations of sperm and concentrations of motile sperm were determined by a Makler counting chamber (Sefi Medical Instruments, Israel) using standard clinical procedures.

Visually assessed sperm motility, which indicates the percentage of sperm with progressive and non-progressive movement (WHO manual 2010) was calculated by using concentration values for sperm and motile sperm. Total numbers of sperm and total numbers of motile sperm were estimated by multiplying the respective concentrations by semen volume, which was measured by pipette. For the assessment of morphology, the criteria of the 2010 WHO Manual were used where the strict criteria < 4% was applied [40]. Anything below this value was considered teratozoospermia. Morphology data were obtained by counting 200 spermatozoa.

If the candidates had greater than 70 million sperm in their ejaculate, they underwent genetic testing, semen culture and blood testing applicable to the donor selection process. Once donors were accepted, if on subsequent donations, there was less than 50 million sperm in the ejaculate, they were excluded from the program. The donors were subsequently underwent psychological evaluations. Sperm donors at our clinic are systematically subjected to the following tests and evaluations: 1) general blood analysis, hemogram, blood group, 2) B and C hepatitis, syphilis, VIH tests 3) cultures to rule out Gonococcus, chlamydia 4) karyotype and systematic investigation of recessive diseases, ruling out candidates who are cystic fibrosis carriers 5) post-thawing sperm survival analysis.

Of the 207 candidates, 43 made it into the donation program. Sperm samples were managed as previously reported [35, 38]. They were then concentrated by volume of ejaculate and centrifuged at 300-400 g for 5–10 min. Donor semen was diluted 1:1 in TEST cryoprotectant medium, and cooled (2–8 ºC) for 45 min in a refrigerator. Finally, a -78 °C CO2 dry ice mould was made and used to make several homogeneous pellets. After 2–3 min they were transferred to a 4.5 mL NUNC cryotube (Sigma Aldrich) and completely immersed in liquid N2 at -196ºC until subsequent thawing. For thawing, the cryotube was placed in a container with water at 37ºC for 15–20 min. The samples were then prepared using Allgrad 45% and Allgrad 90% density gradients (AG 45–050 and AG90-050 LifeGlobal Group, Europe). They were centrifuged for 15 min at 300 g and resuspended in Ferticult culture medium (FertiCult™ IVF medium, FERTIPRO NV, Industriepark Noord 32, B-8730 Beernem, Belgium) for subsequent intrauterine insemination or IVF. The sperm samples were used in 596 AID cycles and in 209 IVF cycles (2120 oocytes).

The patient inclusion criteria for artificial insemination by donor (AID) cycles were all of the following: 1) absence of male partner or ICSI inability/failure because of male factor; 2) woman’s age < 38 years; 3) normal patency assessed by hysterosalpingography; 4) normal uterine cavity assessed by vaginal ultrasound; 5) no evidence of pelvic abnormalities assessed by ultrasound; 6) absence of infectious, metabolic and systemic disorders; 7) body mass index < 37; 8) Anti-Müllerian hormone > 0.4 ng/ml; 9) no suspicion of endometriosis; 10) absence of anovulatory disorders or, if present, good response to gonadotrophin treatment.

The AID method has been reported previously [34]. Briefly, it consisted of the following. Depending on the pregnancy outcome, up to 6 cycles of IUI with one insemination per cycle were performed for all IUI/AID patients. Our protocol consisted of a) modified natural cycle, with ultrasound and estradiol monitoring and hCG triggering, with progesterone luteal phase support or b) ovarian stimulation with 50–100 IU of recombinant FSH (depending on age, AMH, body mass and previous responses) with ultrasound and estradiol monitoring and hCG triggering, with progesterone luteal phase support. The natural cycle was used in the first 3 cycles in women without ovulatory disorders if the age was < 35, whereas stimulation was used in the remaining cases.

The criteria for IVF cycles with a sperm donor were: 1) AID failure; 2) absence of male partner and abnormal tubal patency or age > 38 years; 3) ICSI inability/failure because of male factor. The requirements for IVF-SD cycles were: 1) normal uterine cavity assessed by vaginal ultrasound; 2) absence of infectious, metabolic, systemic disorders; 3) body mass index < 37; 4) Anti-Müllerian hormone > 0.4 ng/ml. Oocyte donation cycles, preimplantation genetic analysis cycles and oocyte accumulation cycles were not included.

The IVF-ICSI method has been reported previously [36, 37]. Briefly, it consisted of the following. A month before ovarian stimulation, contraceptive pills were administered for 10–15 days. If ovarian quiescence was detected, the contraceptive pill was interrupted and 5 days later ovarian stimulation was started. Stimulation was performed with only recombinant FSH in women ≤ 35 years and with recombinant FSH and HMG in women > 35 years. Doses were adjusted depending on age, AMH, body mass and previous responses. In cases with ≤ 12 oocytes, triggering was carried out with rec-hCG, and fresh single embryo transfer was performed on day 5. In cases where ≥ 13 oocytes were expected, triggering was carried out with triptorelin and day 5 single embryo cryotransfer was scheduled. In cryotransfer cycles, endometrial preparation was performed with oral estrogens (6 mg/day) or estrogen patches (150 mg/48 h), and when 7 mm of endometrial diameter and trilaminar phase were achieved, progesterone was started and embryo transfer was performed 5 days later.

The PA status of the donor was unknown to the recipient couples as well as to the attending gynecologists and the biologists selecting the sperm sample, both in AID and IVF.

The IVF laboratory policy with donor sperm consisted of applying conventional IVF in cases with a good expected fertilization rate (woman’s age < 37, no previous fertilization failure or poor fertilization rate, no previous AID failure, and no suspicion of poor oocyte quality). In the remaining cases, ICSI or IVF-ICSI were employed.

## Statistical analysis

The results obtained were checked for normality. Mean/median values were compared by means of ANOVA if they had a normal distribution, or by means of Kruskal–Wallis in non-normal distributions. In the p value comparison by pairs, the Benjamini and Hochberg (1995) correction was applied. Correlation was assessed by means of the Pearson correlation coefficient.

We analyzed separately the influence of the two sports most frequently practiced by our population: running and cycling. Other sports were not analyzed because the number of donor candidates who practiced them was small, precluding their statistical analysis.

The ART outcomes were expressed as follows. Pregnancy was defined as the visualization of the gestational sac on ultrasound performed.For AID, we considered the pregnancy rate (PR); both the global PR (i.e. the number of pregnancies obtained/number of insemination cycles performed) and the mean per-donor PR (i.e. the mean of the quotient number of pregnancies/number of insemination cycles obtained with each donor). For IVF-ICSI we considered the fertilization rate (FR); both the global FR (i.e. the number of oocytes fertilized/ number of oocytes subjected to fertilization) and the mean per-donor FR (i.e. the mean of the quotient number of fertilized oocytes/number of oocytes subjected to fertilization obtained with each donor). Mean rates (AID PR and IVF-ICSI FR) were compared by means of the Student t-test, whereas global rates were compared by means of Pearson χ^2^.

## Results

**Fig 1.**
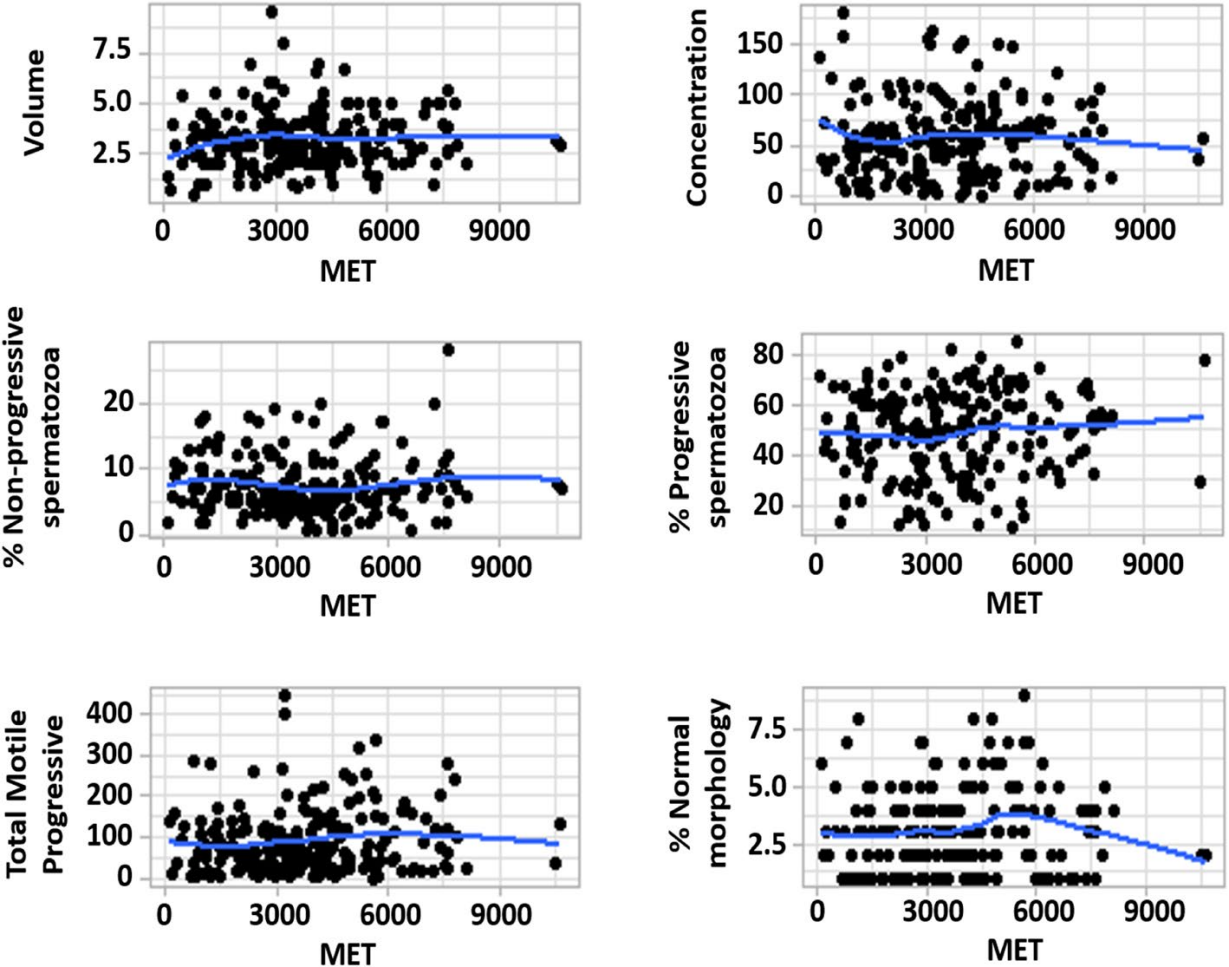
Correlation analysis between physical activity and sperm parameters. No significant correlation. MET = Metabolic equivalent

- 1. Main demographic characteristics (Table 2).
  Age, weight, height and body mass index were very similar in the four groups. There were 6 cases in the low PA group, 73 in the moderate, 101 in the high and 27 in the very high PA group.
- 2. Seminal parameters and PA group (Table 3).
  Sperm parameters did not follow a normal distribution. The low PA group had a slightly higher sperm concentration, but without statistical significance. The group with very high PA had somewhat higher total motile spermatozoa but without statistical significance. There were no significant differences in any of the sperm parameters considered.No correlation was found between PA and any of the analyzed sperm parameters (Fig. 1).
- 3. Specific sports (Table 4).
  Among 35 men reporting cycling, sperm parameters were similar in those who cycled ≤ 1 h per week (n = 18) and in those cycling > 1 h per week (n = 17). When different cut-offs were considered, similar results were obtained (data not shown).Among 66 men reporting running, sperm parameters were similar in those who run ≤ 1 h per week (n = 37) and in those running > 1 h per week (n = 29). When different cut-offs were considered, similar results were obtained (data not shown).There was also no difference in sperm parameters between cyclists and runners.
- 4. Impact of PA in ART (Table 5).
  Of the 207 candidates, 43 were used in our ART program when the study was performed. Of the 6 donors with low PA, none had yet been used at the time of the study. This was due to chance reasons and was not related to either the study design or the policy of our sperm donation program. There were no differences concerning the mean age of the treated women. In AID, the global PR and the mean donor PR were similar in the 3 groups of PA.In IVF/ICSI, the global FRs rates were 62% ± 15.22, 68.55% ± 8.15 and 76.17% ± 13.6 in the moderate, high and very high PA group. Comparing the moderate with the very high PA group and the high with the very high PA group, the differences were close to the significance threshold.When the mean per donor FR was calculated in each PA group, both the very high PA group (72.13%; 264/366) and the high PA group (69.04%; 787/1140) had a significantly higher fertilization rate than the moderate PA group (63.52%; 390/614) (Pearson χ2 = 5.52 and 7.66; p = 0.0056 and 0.018). The main demographic characteristics were very similar in the three PA groups (Suppl table 1).

**Table 2:** Comparison of the demographic characteristics in the four groups of physical activity. (PA).No significant differences.N/A = Not applicable; ns= not significant

	**Low PA **(n= 6)	**Moderate PA **(n= 73)	**High PA **(n= 101)	**Very High PA **(n= 27)	**Normality test**	***p***
**Age (years)**	22.67 ± 5.28	23.11 ± 4.47	23.14 ± 4.95	23.54 ± 4.95	<0.001	ns
**Weight (kg)**	68.17 ± 2.64	72.3 ± 9.02	73.44 ± 9.4	71.59 ± 7.36	0.0179	ns
**Height (m)**	1.76 ± 0.08	1.76 ± 0.05	1.77 ± 0.07	1.76 ± 0.05	0.2795	ns
**BMI (kg/m2)**	22.12 ± 1.68	23.32 ± 3.36	23.28 ± 2.32	23.19 ± 1.69	<0.001	ns
**Glucose (mg/dL)**	97 ± 18.08	88.38 ± 12.7	93.52 ± 12.17	95.94 ± 14.33	<0.001	ns
**Hematocrite (%)**	48.1 ± 4.03	44.52 ± 2.66	44.4 ± 4.76	46.03 ± 3.33	<0.001	ns
**Hemoglobin (g/dL)**	16.27 ± 1.12	14.91 ± 0.93	15.33 ± 3.61	15.09 ± 1	<0.001	ns
**University Students (%)**	50.0 (3/6)	41.1 (30/73)	44.6 (45/101)	40.7 (11/27)	N/A	ns
**Smoking (%)**	16.7 (1/6)	27.4 (20/73)	20.8 (21/101)	18.5 (5/27)	N/A	ns
**Occasional-regular alcohol consumption (%)**	83.3 (5/6)	78.1 (57/73)	85.1 (86/101)	77.8 (21/27)	N/A	ns
**Occasional illicit drug use (%)**	0.0 (0/6)	6.8 (5/73)	7.9 (8/101)	3.7 (1/27)	N/A	ns

**Table 3:** Comparison of the sperm parameters in the four physical activity (PA) groups. No significant differences.Ns= not significant

	**Low PA**	**Moderate PA**	**High PA**	**Very High PA**	**Normality test**	***p***
**Volume (cc)**	2.75 ± 1.75	2.89 ± 1.18	3.3 ± 1.53	3.28 ± 1.26	<0.001	ns
**Concentration (million spermatozoa/cc)**	70 ± 47.02	55.27 ± 43.45	56.73 ± 37.09	57.3 ± 33.61	<0.001	ns
**Progressive spermatozoa (%)**	53.5 ± 13.52	46.79 ± 15.97	47.88 ± 17.03	51.15 ± 16.5	0.0049	ns
**Non-progressive spermatozoa (%)**	7.17 ± 3.43	8.79 ± 4.33	7.13 ± 4.07	8.02 ± 5.08	<0.001	ns
**Total motile progressive (million spermatozoa)**	93.03 ± 59.24	75.63 ± 74.4	85.6 ± 75.55	146.41 ± 295.18	<0.001	ns
**Normal morphology (%)**	3.5 ± 1.64	2.54 ± 1.98	3.03 ± 1.75	3.42 ± 2.02	<0.001	ns

**Table 4:** Sperm parameters among sperm donation candidates according to time devoted to running or cycling. No significant differences

Sport	**Cycling**			**Running**			**Cycling vs Running**		
Time spent on sport per week	**≤ 1h **(n = 18)	**> 1h **(n = 17)	***p***	**≤ 1h **(n = 37)	**> 1h **(n = 29)	***p***	**Cycling**	**Running**	***p***
Volume (cc)	3.28 ± 1.51	3.83 ± 2.02	0.37	3.23 ± 1.34	3.20 ± 1.62	0.94	3.55 ± 1.76	3.22 ± 1.46	0.157
Concentration (million spermatozoa/cc)	58.36 ± 33.98	73.67 ± 46.74	0.27	66.62 ± 42.02	63.23 ± 34.56	0.73	65.80 ± 40.18	65.13 ± 38.74	0.825
Progressive spermatozoa (%)	50.88 ± 15.36	47.28 ± 16.75	0.51	49.45 ± 15.93	49.78 ± 14.97	0.93	49.13 ± 16.04	49.60 ± 15.51	0.78
Non-progressive spermatozoa (%)	6.76 ± 3.13	7.89 ± 4.83	0.42	7.48 ± 4.40	6.59 ± 2.97	0.36	7.31 ± 3.96	7.09 ± 3.77	0.808
Total motile progressive (million spermatozoa)	104.04 ± 90.41	118.39 ± 93.64	0.65	102.32 ± 74.75	91.56 ± 60.14	0.53	111.01 ± 91.98	97.59 ± 68.33	0.547
Normal morphology (%)	3.07 ± 1.91	2.89 ± 1.64	0.78	3.25 ± 1.60	3.25 ± 2.01	1.00	2.98 ± 1.78	3.25 ± 1.78	0.147

**Table 5:** Pregnancy rates obtained in artificial insemination donors and IVF/ICSI according to the physical activity of the donors. Significant differences in mean donor fertilization rate in ICSI/IVF (%) when comparing moderate PA with high PA and moderate PA with very high PA. No significant differences in the mean age of the treated women.* = comparison of Moderate PA vs High PA; ** = comparison of Moderate PA vs Very High PA;*** =comparison of High PA versus Very High PA

	**Mean per donor fertilization rate in ICSI/IVF (%)**	**High PA **(n= 22)	**Very High PA **(n= 6)	**P**
**Global AID pregnancy rate (%) **	24.24 ± 10.10	25.5 ± 13.14	13.82 ± 13.53	*0.900; **0.360; ***0.220
**Mean donor pregnancy rate in AID (%)**	23.47 (50/213)	24 (66/275)	20.3 (22/108)	*0.890; **0.520; ***0.440
**Global IVF/ICSI fertilization rate (%)**	62.0 ± 15.22	68.55 ± 8.15	76.17 ± 13.6	*0.110; **0.060; ***0.099
**Mean per donor fertilization rate in ICSI/IVF (%)**	63.52 (390/614)	69.04 (787/1140)	72.13 (264/366)	*0.018; **0.006; ***0.260

## Discussion

There is general agreement regarding the beneficial health effects of PA on health [1, 29]. However, there is some concern regarding the effects of very intense PA on health. Thus a number of conditions have been associated with very intense PA, such as risk of atrial fibrillation, coronary artery disease, and malignant ventricular arrhythmias, sudden cardiac death [28, 43] and orthopedic overuse injuries [44]. It has been reported that light/moderate PA increases life expectancy, but people who chronically log very high doses of vigorous PA appear to lose most or all of the protection against early mortality and CV [43].

In the case of male fertility and PA, it is not clear whether there is an optimal level of PA and if some patients should be encouraged to increase or decrease their PA. In a recent meta-analysis [21] it was shown that both mean-intensity recreational activity and high-intensity recreational activity were associated with better sperm quality. On the other hand, in elite athletes physical activity was correlated with a decline in sperm quality [22].

Our study did not show any adverse effects of high PA, even of very high PA, on sperm quality. Moreover, multivariate analysis showed no correlation of PA with sperm parameters.

Our results are in agreement with those obtained in a study performed also in Spain, in volunteer university students [41]. However, our results disagree with those of a recent study performed in China, also in sperm donors [56] where the increase in PA was associated with better sperm quality. We think that the discrepancies may be due to the differences in sample population, both ethnic and geographical as well as age and sample obtainment. Our donors were all Caucasian, their mean age was 24.5 (compared with 28.5 in Sun’s report) and the mean abstinence time was 4 days (vs 6.2 in Sun). Moreover, it has to be highlighted that the proportion of donor candidates with low PA was very small in our population. Thus, in our population only 2.9% (6/207) of participants had < 600 METs, whereas in Sun’s study the median of METs in the first quartile was 526.

Our study has some limitations. On the one hand, we have studied only one sample per donor, and variability in sperm parameters is well known [8, 10]. Although from an individual point of view more than one sperm analysis should be performed, there appear to be limited advantages to using more than one semen sample in epidemiological studies [7, 41, 55]. Additionally, PA was measured by means of the IPAQ questionnaire, which has its limitations, as with all questionnaires. However, the IPAQ has shown a good correlation with the PA measured by accelerometers [12, 45] and it has also been validated in our population [50]. Although a measurement error cannot be discarded, the measurement errors, if any, are more likely to be non-differential (i.e. unrelated to the semen quality parameters). On the other hand, since our study was observational, a number of confounding factors cannot be ruled out, such as social status, economic income, health preoccupation, health status and ethnic background, which could have had an impact on sperm quality. However, all our sperm donors were Caucasians, and of a similar social background. Moreover, they were young, almost all with normal weight (98% BMI 19.5–25) and without health problems. We have to highlight that in our area the obesity rate is relatively low (4.9% of males aged 15–29) [17]. Furthermore, very high PA may be associated with the intake of illicit drugs, which could impair spermatogenesis; in our study, the preselection of the donors included a thorough interview and if illegal drugs were reported, they were excluded as donors. Moreover, in any case, if in some case illegal drug consumption – associated with very high PA – was not reported, it would impair seminal quality, which was not the case in our study, since in high PA group sperm parameters were not impaired. Our study was performed in a young healthy population without fertility problems. Thus, results might not be the same in a different population. However, our results match exactly with those obtained with a similar methodology in the very same geographical area but in males from infertile couples (mean age close to 38 years): neither low nor very high PA activity was associated with higher or lower semen quality [21] It has to be stressed that none of our donor candidates were elite athletes, and thus our results may not be extrapolated to this particular demographic.

Our study has the following strengths. The study only recruited healthy young men, whose previous fertility was unknown, and therefore a number of confounding factors were avoided. Moreover, all the potential sperm donors come from the same geographical area, which is relatively small and thus many environmental factors should be controlled. Moreover, various different sports were inquired about, the most common of which was running. In agreement with some previous reports [22], the time dedicated to running did not have an impact on sperm quality. On the other hand, it has been reported that cycling could be one of the most sperm-damaging sports [5, 26]. In patients attending an infertility clinic, impaired seminal parameters have been reported among those cycling ≥ 1.5 h per week [13] and in another, ≥ 5 h per week [62], whereas in another study a non-significant trend was reported [21] However, in our study no differences in sperm parameters were observed when different subgroups in cycling activity were considered, nor when cyclists were compared with runners. However, it must be taken into account that only one sample per potential sperm donor was studied, and that the use of questionnaires always involves a certain degree of inaccuracy.

As a marker of sperm quality in the men finally accepted as sperm donors at our clinic, our study included the fertility parameters obtained in IVF and AID cycles. In couples where the woman has a normal fertility work-up and undergoes a spontaneous natural cycle, PR is one of the best, if not the best, markers of sperm quality. However, with ART techniques there is some compensation for the reduced fertilization ability of the sperm, especially with ICSI where the fertilization rates obtained with poor quality sperm are very similar to those obtained with normal sperm. Although it is well known that with sperm samples under a cutoff, ART results are lower, it is controversial whether the increase in sperm parameters produces better results above the normality cutoff [31, 47].

Our ART analysis, however, seems paradoxical. On one hand in AID, the per-cycle PR was very similar in the different PA subgroups. On the other hand, IVF/ICSI fertilization rates were significantly higher in high and very high PA groups compared with moderate PA group. No comparison could be made with low PA since no sample from donors with low PA was employed. In our opinion, this higher FR might reflect better sperm functionality associated with PA which could not be shown in AID, since AID PR is influenced by many female parameters (stimulation protocol/natural cycle, mild undetected tubal conditions, endometrial receptivity), whose influence on IVF FR is much lower. The most important female factor in IVF fertilization is the woman’s age, and this was very similar in the 3 groups considered. Notwithstanding, our results should be interpreted with caution, since the ART population was relatively small and variable in regard to other female characteristics and stimulation protocols utilized.

Although the use of two different fertilization techniques could have influenced the outcome of the fertilization rates, it should be noted that the choice of one or the other was based on maternal criteria (such age and previous fertilization) and not on seminal parameters. Given that the SDs were chosen randomly, without taking into account the fertility characteristics of the recipient, it would not be expected that a differential bias in the results would be attributed to the use of one or the other fertilization technique. On the other hand, although there is controversy, reproductive outcomes appear to be similar with IVF or ICSI using standard sperm [6, 30, 51].

From our study, we can conclude that in sperm donor candidates sperm parameters were not influenced by PA, even in high and very high PA groups. More studies are needed to investigate the better fertilization rates associated with high and very high PA in IVF cycles. In our opinion, from the male fertility point of view for the time being, there is no evidence to give recommendations regarding increasing or decreasing PA in order to improve sperm quality.

## Supplementary Information

**Additional file 1: Table 1.** Characteristics of the women undergoing artificial insemination or IVF treatment expressed as mean (SD) or percentage. No significant differences were found between groups of different physical activity. Parameters of age, body mass index, antiMüllerian value or smoking were compared. Patients with endometriosis had no indication for AID.

## Data Availability

Not applicable.
