# Supplementary material for: Physical activity and sperm quality: influence in sperm donors
Source: Reprod Biol Endocrinol. 2022 May 24;20:83. doi: 10.1186/s12958-022-00946-x (PMC9128101; doi:10.1186/s12958-022-00946-x)
Supplement: Supplementary file 1 — Additional file 1: Table 1. Characteristics of the women undergoing artificial insemination or IVF treatment expressed as mean (SD) or percentage. No significant differences were found between groups of different physical activity. Parameters of age, body mass index, antiMüllerian value or smoking were compared. Patients with endometriosis had no indication for AID. [file 12958_2022_946_MOESM1_ESM.docx]

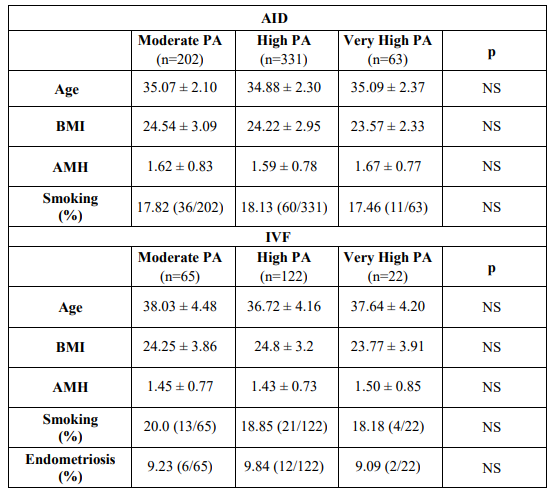


**Supplementary table 1.** Characteristics of the women undergoing artificial insemination or IVF treatment expressed as mean (SD) or percentage. No significant differences were found between groups of different physical activity. Parameters of age, body mass index, antiMüllerian value or smoking were compared. Patients with endometriosis had no indication for AID.
